# Central and peripheral pulmonary sclerosing pneumocytomas: multi-phase CT study and comparison with Ki-67

**DOI:** 10.2478/raon-2023-0042

**Published:** 2023-09-04

**Authors:** Yanli Zhang, Chao Ran, Wei Li

**Affiliations:** Department of Clinical Pharmacy, Affiliated Hospital of Yangzhou University, Yangzhou, China.; Department of Radiology, Affiliated Yantai Yuhuangding Hospital of Qingdao University, Yantai, China; Department of Medical Imaging, Affiliated Hospital of Yangzhou University, Yangzhou, China.

**Keywords:** pulmonary sclerosing pneumocytoma, location, multi-phase computed tomography, Ki-67

## Abstract

**Background:**

This study aimed to evaluate the multi-phase CT findings of central and peripheral pulmonary sclerosing pneumocytomas (PSPs) and compared them with Ki-67 to reveal their neoplastic nature.

**Patients and methods:**

Multi-phase CT and clinical data of 33 PSPs (15 central PSPs and 18 peripheral PSPs) were retrospectively analyzed and compared their multi-phase CT features and Ki-67 levels.

**Results:**

For quantitative indicators, central PSPs were larger than peripheral PSPs (10.39 ± 3.25 cm^3^ *vs.* 4.65 ± 2.61 cm^3^, P = 0.013), and tumor size was negatively correlated with acceleration index (r = −0.845, P < 0.001). The peak enhancement of central PSPs appeared in the delayed phase, with a longer time to peak enhancement (TTP, 100.81 ± 19.01 s), lower acceleration index (0.63 ± 0.17), progressive enhancement, and higher Ki-67 level. The peak enhancement of peripheral PSPs appeared in the venous phase, with the shorter TTP (62.67 ± 20.96 s, P < 0.001), higher acceleration index (0.99 ± 0.25, P < 0.001), enhancement washout, and lower Ki-67 level. For qualitative indicators, the overlying vessel sign (86.67% *vs.* 44.44%, P = 0.027), prominent pulmonary artery sign (73.33% *vs.* 27.78%, P = 0.015), and obstructive inflammation/atelectasis (26.67% *vs.* 0%, P = 0.033) were more common in central PSPs, while peripheral PSPs were more common with halo sign (38.89% *vs.* 6.67%, P = 0.046).

**Conclusions:**

The location of PSP is a possible contributing factor to its diverse imaging-pathological findings. The tumor size, multi-phase enhancement, qualitative signs, and Ki-67 were different between central and peripheral PSPs. Combined tumor size, multi-phase findings, and Ki-67 level are helpful to reveal the nature of the borderline tumor.

## Introduction

Pulmonary sclerosing pneumocytoma (PSP), formerly known as pulmonary sclerosing hemangioma, is a rare pulmonary lesion, which was first described by Liebow and Hubbell in 1956.^1^ PSP is most common in middle-aged women in East Asia, accounting for 3–5% of benign lung tumors.^2^ Most patients are asymptomatic and clinically incidental. Some studies suggested that the symptoms and surgical methods of PSP were related to tumor location and its mass effect. Larger lesions near the hilum may be more likely to cause respiratory symptoms.^3^ Lobectomy is often performed for central lesions, while enucleation or wedge resection is more common for peripheral lesions, without systematic lymph node dissection and subsequent radiochemotherapy.^4^ However, the influence of tumor location on PSP imaging has not been specifically evaluated. Moreover, the neoplastic nature of PSP is still controversial.^5^ Althoug h PSP is considered a benign tumor with a good prognosis, it originates from the primitive respiratory epithelium with possible metastasis and recurrence.^6,7^ The intraoperative frozen biopsy of PSP is easily confused with adenocarcinoma or carcinoid, and the lower Ki-67 level has a certain discriminative effect.^8,9^ As a marker of cellular proliferation and malignant potential, Ki-67 is almost not expressed in normal tissues but is significantly elevated in various malignant tumors, especially lung cancer.^9^ Its expression gradually increases with the tumoral occurrence, growth, and metastasis.^10^ For example, the Ki-67 index of non-small cell lung cancer is often more than 60%, indicating high malignancy, rapid progression, and poor prognosis.^11^ While the Ki-67 level of adenocarcinoma is lower than other types of lung cancer, which may be related to its lower proliferation.^10^ On medical imaging, PSP has the morphological characteristics of benign lesions, but with stronger enhancement on CT and higher fluorodeoxyglucose accumulation on PET/CT.^12,13,14^ Compared with benign pulmonary tumors (hamartomas), the enhancement of PSP is more obvious, with less calcification and fat component.^12,13^ Compared with malignant pulmonary tumors (adenocarcinomas or carcinoid tumors), the enhancement duration of PSP is longer, with rare lobulation and pleural invasion.^12,13^ All these findings obscure its neoplastic nature. Dual-phase arteriovenous enhancement may miss some diagnostic information. While multi-phase CT can more fully show the evolution of PSP enhancement, and achieve a similar effect to dynamic enhancement with less radiation exposure. To enrich the diagnostic information of PSP, the multi-phase CT findings of central and peripheral PSPs were analyzed retrospectively, and compared with the Ki-67 to provide new insights into their neoplastic nature.

## Patients and methods

### Patients

This retrospective study was designed and conducted in accordance with the Declaration of Helsinki and was approved by the ethics and review board of Affiliated Yantai Yuhuangding Hospital of Qingdao University. Written informed consent was obtained from all the study participants. Thirty-three patients with PSP confirmed by pathology from 2016 to 2022 were collected, and their clinical and imaging data were retrospectively analyzed. The pathological diagnosis was determined by cell morphology and immunohistochemistry. Inclusion criteria: complete clinical data, definite pathological diagnosis, and consistent imaging protocols. Exclusion criteria: transthoracic needle aspiration biopsy before multi-phase CT examination (avoiding the influence of intratumoral hemorrhage on CT enhancement), concomitant with other pulmonary tumors, and lesions too small to measure the CT density accurately.

### Imaging examinations

GE Optima CT660 (GE Healthcare, Milwaukee, USA) was used in all patients for multi-phase scanning (tube voltage = 120 kV, tube current = 10–300 mA, collimation width = 0.625 mm×128, spiral pitch = 1:1, slice thickness = 5 mm, slice interval = 5 mm), including unenhanced, arterial, venous and delayed phases. According to the different weight and cardiovascular status, the contrast medium was administrated at 3.0–3.5 mL/s and 1.5–2.0 mL/kg. After intravenous injection of a non-ionic contrast agent (Iohexol Injection, 300 mg I/mL), the arterial phase scanning was delayed for 20–25 s, the venous phase scanning was delayed for 45–65 s, and the delayed phase scanning was delayed for 95–120 s.

### Imaging analysis

Imaging evaluation was performed on the Medcare AnyImage workstation (V4.5). Reconstruction was performed with a thickness of 1 mm to reduce partial volume artifact, and multiple image post-processing techniques were performed to determine the tumor locations. The tumor size was measured on lung-window images (window width, 1000 – 2000 Hu; window level, −800 – −450 Hu), and the tumor density was measured on mediastinal-window images (window width, 250 – 500 Hu; window level, 30 – 50 Hu). Based on the location of the lesion, the patients in this study were divided into central PSPs (15 cases, lesions were near the hilum and adjoined the primary or secondary bronchus) and peripheral PSPs (18 cases, lesions were near the chest wall and located distal to segmental bronchus). Imaging observation included quantitative and qualitative indicators. Quantitative indicators: tumor size (mm^3^), multi-phase (unenhanced, arterial, venous, and delayed phases) CT densities (Hounsfield unit, Hu), peak enhancement value (a maximum density in the enhancement duration, Hu), net enhancement value (peak enhancement – unenhanced density, Hu), time to peak enhancement (TTP, second), enhancement washout value (peak enhancement – delayed phase density, Hu) and acceleration index (net enhancement / TTP). When measuring the CT density of each phase, the region of interest (ROI) should be set at the same slice, covering the central part of the tumor as much as possible, avoiding possible cystic change and hemorrhage, and then the average CT density of three different slices should be taken. Qualitative indicators: tumor morphology, obstructive inflammation/atelectasis, overlying vessel sign, prominent pulmonary artery sign, and halo sign. The overlying vessel sign referred to the compressed vessel around the lesion. A prominent pulmonary artery sign was defined as the obvious enlargement of the pulmonary artery adjacent to the lesion, compared with the contralateral similar pulmonary artery. The ground-glass opacity around the lesion was considered as the halo sign. All imaging data were evaluated by two chest radiologists with ten years of diagnosis experience and without knowledge of pathological findings. In case of any disagreement, it shall be settled through consultation.

### Statistical analysis

In this study, descriptive statistics were processed by IBM SPSS version 22.0 (SPSS, Chicago, IL, USA). All data were presented as numbers (percentage) or mean ± SD. An independent t-test (two-tailed) or Mann-Whitney U-test was used to assess the difference between continuous variables. The Chi-square test or Fisher's exact test (two-tailed) was used for the statistical comparison of dichotomous variables. Pearson correlation was analyzed between the tumor size and acceleration index. A p-value of < 0.05 was defined as statistically significant. The inter-observer variability was examined by the interclass correlation coefficient (ICC) or Kappa value. ICC > 0.75 or Kappa ≥ 0.8 was considered a better agreement.

## Results

### Clinical findings

**FIGURE 1.**
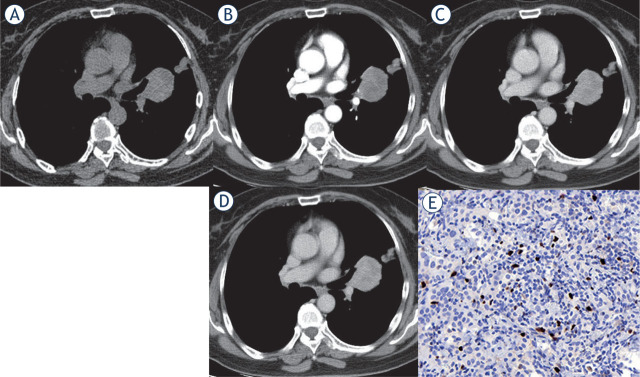
Multi-phase contrast-enhanced CT of central PSP with higher Ki-67 index. Axial unenhanced CT image revealed a roundish isodense lesion in the left perihilar region with a CT density of 49 Hu **(A)**. After administration of contrast medium, the lesion showed inhomogeneous enhancement with the enlarged left inferior pulmonary artery and overlying vessel sign **(B)**. The lesions showed progressive and continuous enhancement in the arterial phase (75 Hu) **(B)**, venous phase (96 Hu) **(C)**, and delayed phase (110 Hu) **(D)**. Immunohistochemical staining showed the Ki-67 reactive tumor cells accounted for about 10% (× 400) **(E)**.

**FIGURE 2.**
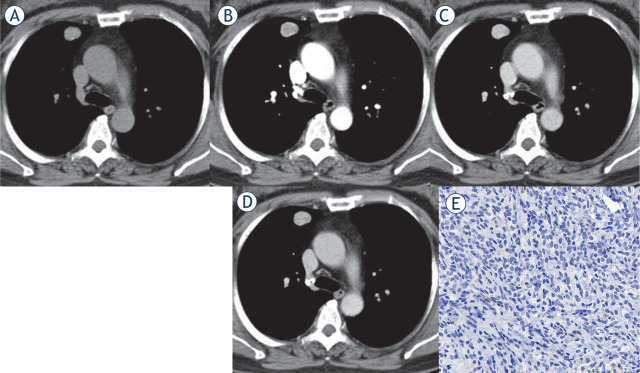
Multi-phase contrast-enhanced CT of peripheral PSP with lower Ki-67 index. Axial unenhanced CT image revealed a peripheral isodense nodule in the right upper lobe with a CT density of 46 Hu **(A)**. After administration of the contrast medium, the lesions showed homogeneous enhancement. The lesion showed progressive and continuous enhancement in the arterial phase (73 Hu) **(B)** and venous phase (102 Hu) **(C)**, with a certain enhancement washout in the delayed phase (87 Hu) **(D)**. Immunohistochemical staining showed the Ki-67 reactive tumor cells accounted for about 1% (× 400) **(E)**.

All 33 patients were female, with an average age of 54.1 ± 8.2 years. Most of them were found incidentally in healthy examinations (23 cases, 69.7%), and accompanied by non-specific respiratory symptoms (cough, expectoration, chest discomfort, fever, etc.). Compared with peripheral PSP, these non-specific respiratory symptoms were more common in central PSP (73.33% *vs*. 33.33%, P = 0.037). Both groups of PSP patients underwent surgical treatment, with a similar age of onset (56.6 ± 8.5 years *vs.* 51.6 ± 7.7 years, P = 0.244). Patients with central PSP were treated with lobectomy, while those with peripheral PSP underwent enucleation or wedge resection. No lymph node metastasis or surrounding structural invasion was found during the operation. Histopathologically, polygonal cells and surface cubic cells constituted hemangiomatous, papillary, sclerotic, and solid regions in different proportions. Immunohistochemically, thyroid transcription factor-1 (TTF-1, +), synapsin (Syn, −), epithelial membrane antigen, (EMA, +), and carcinoembryonic antigen (CEA, −) were shown in both tumor cell types. The Ki-67 index of all PSPs did not exceed 10%, most of them (26 cases, 78.79%) were no more than 3% and the others (7 cases, 21.21%) were 3–10%. The lower Ki-67 level (Ki-67 index ≤ 3%) was more common in peripheral PSP than in central PSP (94.44% *vs*. 60%, P = 0.030). None of the patients received any postoperative radiochemotherapy, and no recurrence or progression has been observed in the follow-up (1–7 years). See Table 1, and Figures 1–2 for details.

**TABLE 1.** Imaging and clinical comparisons between central and peripheral pulmonary sclerosing pneumocytomas (PSPs)

	**Central PSPs (n = 15)**	**Peripheral PSPs (n = 18)**	**P**
Age (years)	56.6 ± 8.5	51.6 ± 7.7	0.244
Size (cm^3^)	10.39 ± 3.25	4.65 ± 2.61	0.013^*^
Respiratory symptoms			
n, present:absent	11:4	4:14	0.037^*^
Unenhanced CT density (Hu)	37.57 ± 15.61	43.64 ± 13.09	0.312
Arterial phase CT density (Hu)	67.09 ± 16.99	69.79 ± 18.67	0.767
Venous phase CT density (Hu)	91.36 ± 20.43	97.14 ± 21.38	0.373
Delayed phase CT density (Hu)	98.73 ± 26.53	74.71 ± 24.97	0.044^*^
Net enhancement value (Hu)	61.47 ± 12.18	57.11 ± 10.28	0.205
Peak enhancement value (Hu)	98.73 ± 26.53	97.14 ± 21.38	0.828
Enhancement washout value (Hu)	3.8 ± 8.14	20.78 ± 10.22	< 0.001^*^
TTP (s)	100.81 ± 19.01	62.67 ± 20.96	< 0.001^*^
Accelerated index	0.63 ± 0.17	0.99 ± 0.25	< 0.001^*^
Ki-67 index			
n, low:high^#^	9:6	17:1	0.030^*^
Overlying vessel sign			
n, present:absent	13:2	8:10	0.027^*^
Prominent pulmonary artery sign			
n, present:absent	11:4	5:13	0.015^*^
Obstructive inflammation/atelectasis			
n, present:absent	4:11	0:18	0.033^*^
Halo sign			
n, present:absent	1:14	7:11	0.046^*^
Peak phase			
n, venous phase:delayed phase	3:12	15:3	< 0.001^*^

Accelerated index = net enhancement/TTP; Hu = Hounsfield unit; PSP = pulmonary sclerosing pneumocytoma; TTP = time to peak enhancement; Values are given as n (= number) or mean ± SD

* Significance values;

# Low = Ki-67 index ≤ 3%; High = Ki-67 index > 3%

### Imaging findings

For these quantitative and qualitative CT analyses, good inter-observer agreements were obtained between the two observers (ICC = 0.8871, Kappa = 0.8692).

A total of 33 solitary lesions were found in this study. The size of 33 PSP lesions was negatively correlated with the acceleration index (r = −0.845, P < 0.001). The central lesions were larger than the peripheral lesions (10.39 ± 3.25 cm^3^ *vs.* 4.65 ± 2.61 cm^3^, P = 0.013). There was no difference in CT densities of unenhanced, arterial, and venous phases between the two groups, but the delayed enhancement of central PSPs was more obvious than that of peripheral PSPs (98.73 ± 26.53 Hu *vs.* 74.71 ± 24.97 Hu, P = 0.044). There was no difference in peak enhancement and net enhancement values. The peak enhancement of central PSPs appeared in the delayed phase (12/15, 80%), with a longer time to peak enhancement (TTP, 100.81 ± 19.01 s), lower acceleration index (0.63 ± 0.17), and progressive enhancement. The peak enhancement of peripheral PSPs appeared in the venous phase (15/18, 83.33%, P < 0.001), with the shorter TTP (62.67 ± 20.96 s, P < 0.001), higher acceleration index (0.99 ± 0.25, P < 0.001) and delayed enhancement washout (20.78 ± 10.22 Hu *vs.* 3.8 ± 8.14 Hu, P < 0.001).

Both types of PSP lesions were roundish with smooth edges. Compared with peripheral PSPs, obstructive inflammation/atelectasis (26.67% *vs.* 0, P = 0.033), overlying vessel sign (86.67% *vs.* 44.44%, P = 0.027), and prominent pulmonary artery sign (73.33% *vs.* 27.78%, P = 0.015) were more common in central PSPs. While peripheral PSPs were more common with a halo sign than central PSPs (38.89% *vs.* 6.67%, P = 0.046). See Table 1, and Figures 1–2 for details.

## Discussion

PSP was initially considered a variant of hemangioma, with an obvious tendency of angiogenesis and sclerosis.^15^ In 2015, the World Health Organization (WHO) changed its classification from “miscellaneous tumors” to “adenomas”.^16^ Due to the nonspecific clinical symptoms, most patients with PSP in this study were found by healthy examination, and its female susceptibility might be associated with estrogen and progesterone.^17^ The larger central PSP is adjacent to the hilum, making it more likely to compress the proximal bronchi, resulting in more respiratory symptoms and obstructive inflammation/atelectasis. The mutual migration and coexistence of four histological regions make it difficult to confirm the predominant component of PSP with limited pathological sampling.^18^ The ki-67 index is no more than 3% in normal cells and more than 10% in malignant tumors.^9,19^ However, the Ki-67 index was 3–10% in 7 cases (21.21%) of PSP in this study. The diverse Ki-67 expression, TTF-1 (+), and EMA (+) suggested that PSP originated from the primitive alveolar epithelium and with a certain growth potential.

Because central PSPs were adjacent to the pulmonary hilar vessels, it was easier to get sufficient blood supply and tumor growth.^20^ Therefore, in this study, the central PSPs were larger than the peripheral PSPs. The size of PSP was also thought to be related to Ki-67, representing tumoral proliferation. The larger PSP contains more pure malignant components with a higher KI-67 level.^21^ Although the central PSPs were larger than the peripheral PSPs and with the higher Ki-67 level, there was no difference in peak enhancement and net enhancement between them. The mixture of pathological components might offset the enhancement differences.^22,23^ In addition, the size of PSPs was negatively correlated with the acceleration index. With a similar net enhancement, the shorter TTP of smaller peripheral PSPs resulted in a higher perfusion efficiency and accelerated index, which were more common in malignant lesions.^22,24^

The central and peripheral PSPs had similar isodensity on unenhanced CT, which provided the comparability for subsequent contrast enhancement. The central and peripheral lesions showed continuous enhancement at the arterial and venous phases, which was consistent with the characteristic enhancement of PSP.^25^ However, these PSPs showed different enhancements during the delay phase. This highlights the superiority of multi-phase CT scanning. The histological evolution of PSP might not always follow the hemangiomatous-papillary-solid-sclerotic sequence.^26,27^ With the growth of PSP, its enhancement characteristics changed according to the tumor size and its pathological components.^27,28^ The smaller peripheral PSPs mainly contained papillary and hemangiomatous components and were enhanced significantly in arterial and venous phases, with a peak enhancement in the venous phase.^29,30^ While the enhancement washout in the delayed phase was a possible malignant sign.^22,24^ The larger central PSPs contain more complex tumoral components (mainly sclerotic and solid components), resulting in continuous enhancement during the delayed phase.^29,30^ This showed a progressive enhancement of benign tumors.

A comprehensive analysis of tumor size, multi-phase enhancement, and Ki-67 of central and peripheral PSPs is helpful to understand their tumor nature. The Ki-67 level of larger central PSP was higher, but it showed progressive enhancement, longer TTP, and lower accelerated index (benign imaging feature). While the Ki-67 level of smaller peripheral PSP was lower, with the enhancement washout, shorter TTP, and higher accelerated index (malignant imaging feature). The inconsistency between Ki-67 and enhancement mode further revealed the nature of borderline tumors with some malignant potential. Besides, the different enhancements of central and peripheral PSPs might also be associated with the CT phase setting. Multi-phase CT (arterial, venous, and delayed phases) could extensively cover the microperfusion of PSP, which was conducive to displaying the various tumor components and avoiding the omission of diagnostic information to the greatest extent.

Overlying vessel sign is caused by the compressed blood vessels around the PSP lesions, reflecting the growing tendency of neighbor vessels to the tumors.^31^ Previous studies showed that overlying vessel sign was more common in peripheral lesions.^30,31^ However, similar to prominent pulmonary artery signs, the overlying vessel sign was more common in central PSPs in this study. This may be due to the larger central PSPs being closer to the pulmonary hilar vascular branches.^25,29^ Compared to intratumoral microvessels, the larger extratumoral vessels are unlikely to affect the enhancement degree of PSP.^24^ Therefore, there was no difference in the degree of enhancement between the central and peripheral PSPs. Peripheral PSPs were more likely to compress small airways, causing distal bronchial obstruction or local pulmonary congestion, and then the halo sign was common.^32^

Due to the limitation of sampling and irregular histological distribution, it is difficult to match the multi-phase enhancement and pathological components precisely.^14^ We employ intelligent radiation dose tracking technology to minimize additional radiation exposure from multi-phase CT scans. A single-center retrospective study is difficult to avoid selection bias. The small sample size due to low incidence limited the statistical reliability. Further multi-center study with larger samples is necessary. Indeed, a single tumor marker cannot fully reflect the neoplastic essence. In clinical applications, it is important to combine Ki-67 with multi-phase CT for the evaluation of other tumors.

## Conclusions

The locations of PSP may lead to differences in lesion size, multi-phase enhancement, qualitative CT signs, and Ki-67, which deepens our understanding of PSP. The central PSP is larger and has a higher Ki-67 level but with progressive enhancement, longer TTP, and a lower acceleration index. The peripheral PSP is smaller and has a lower Ki-67 level but with a shorter TTP, higher acceleration index, and enhancement washout. Combining these multi-phase CT features with the Ki-67 level can clarify the borderline nature of PSP. Furthermore, when Ki-67 is further elevated, the possibility of other pulmonary malignancies should be considered.
